# The effect of vitamin D supplementation on oxidative stress and inflammatory biomarkers in pregnant women: a systematic review and meta-analysis of clinical trials

**DOI:** 10.1186/s12884-022-05132-w

**Published:** 2022-11-05

**Authors:** Soudabe Motamed, Bahareh Nikooyeh, Razieh Anari, Somayeh Motamed, Zeinab Mokhtari, Tirang Neyestani

**Affiliations:** 1 Asadabad School of Medical Sciences, Asadabad, Iran; 2 National Nutrition and Food Technology Research Institute and Faculty of Nutrition Sciences and Food Technology, Shahid Beheshti University of Medical Sciences, Tehran, Iran; 3 Tehran University of Medical Sciences, School of Medicine, Tehran, Iran; 4 Food Security Research Center, Isfahan University of Medical Sciences, Isfahan, Iran

**Keywords:** Vitamin D, Pregnancy, Inflammation, Oxidative stress, Meta-analysis

## Abstract

**Background:**

Vitamin D deficiency, a common problem among pregnant women, is linked with maternal inflammation, oxidative stress and consequent adverse pregnancy outcomes. The aim of this systematic review was to evaluate the effect of vitamin D supplementation on oxidative stress and inflammatory biomarkers in pregnant women according to the PRISMA guidance.

**Methods:**

Four databases including PubMed/MEDLINE, Scopus, Web of Science and Cochrane were used for searching papers published until 25^th^ July 2022. Clinical trials that assessed 25-Hydroxyvitamin D (25(OH)D), inflammatory markers (including high sensitivity C-reactive protein (hs-CRP) and certain cytokines) and oxidative stress markers (including malondialdehyde (MDA), total antioxidant capacity (TAC) and glutathione (GSH)) in pregnant women, were included in this review. The primary search of three databases displayed 21571 records. After removing duplicates and irrelevant articles, 17 eligible RCTs included for more evaluation. Random effect model and Der Simonian-Laird method were used to pool the data of studies. Risk of bias assessed according to version 2 of the Cochrane risk-of-bias tool for randomized trials.

**Results:**

According to the meta-analysis result, vitamin D supplementation caused a significant increase in the maternal circulating concentrations of 25(OH)D (SMD 2.07; 95%, CI 1.51, 2.63; *p* < 0.001), TAC (SMD 2.13, 95% CI 1.04 to 3.23, *p* < 0.001) and GSH (SMD 4.37, 95% CI 2.9 to 5.74, *p* < 0.001) as well as a significant decrease in the levels of MDA (SMD -0.46, 95% CI -0.87 to -0.05, *p* = 0.02). However, it had no significant impact on hs-CRP concentrations (SMD 0.24; 95% CI, -0.55, 1.03; *p* = 0.50).

**Conclusion:**

In the present study, vitamin D supplementation led to increased levels of 25(OH)D, TAC and GSH and also decreased concentration of MDA. Nevertheless, because of low certainty of evidence, these findings have to be declared conservatively.

**Trial registration:**

Registration code in PROSPERO website: CRD42020202600

**Supplementary Information:**

The online version contains supplementary material available at 10.1186/s12884-022-05132-w.

## Introduction

Vitamin D deficiency which is well-known as the world public health problem is highly prevalent among pregnant women and their infants who are more vulnerable than any other population groups. Suboptimal vitamin D status not only do cause bone diseases but it also result in immune dysfunction and the predominance of pro-inflammatory status and production of harmful free radicals [1, 2].

Recent evidence indicates that maternal inflammation usually accompanied by augmented oxidative stress may result in the adverse pregnancy outcomes including gestational diabetes mellitus (GDM), hypertension disorders, spontaneous abortion, restricted fetal growth, small for gestational age (SGA), pre-term delivery, low birth weight (LBW) and fetal impaired neuronal development [3–6]. Accumulating data have supported the unfavorable effect of hypovitaminosis D during pregnancy on detrimental outcomes as a result of amplified inflammatory state and oxidative stress [7, 8]. Instead, clinical trials demonstrated that optimizing maternal vitamin D status can relief this situation through its immunomodulatory properties [9–12].

It has been suggested that vitamin D modulates immune system by down-regulation of pro-inflammatory cytokines like interleukin (IL)-6, IL-1, tumor necrosis factor-alpha (TNF-α) and high sensitivity C reactive protein (hs-CRP) and also by up-regulation of anti-inflammatory cytokines such as IL-4 and IL-10 [13–15]. Nevertheless, because of the sophisticated physiology of pregnancy, the overall effect of vitamin D on the inflammatory and oxidative stress biomarkers during pregnancy has not been clarified yet.

So far, a few clinical trials have been conducted among pregnant women to investigate the association between vitamin D status and inflammation and oxidative stress as plausible determinants of the occurrence of adverse pregnancy outcomes. Therefore, the aim of this study was to fill the knowledge gap regarding the effect of vitamin D on biomarkers of inflammation and oxidative stress during pregnancy through a systematic review and meta-analysis of the randomized controlled clinical trials (RCTs).

## Methods

In this systematic review and meta-analysis, the standard of the PRISMA (Preferred Reporting Items for Systematic Reviews and Meta-analyses) was employed. The protocol of this study has been registered in PROSPERO website (code: CRD42020202600 available at: https://www.crd.york.ac.uk/prospero/display_record.php?ID=CRD42020202600).

### Search strategy

A comprehensive literature search was conducted through electronic databases including PubMed, Scopus, Web of Science and Cochrane for papers published from inception to 25^th^ July 2022 without language restriction. The search terms for electronic databases used combinations of *pregnancy, gestation, child bearing, gravidity, intrauterine pregnancy, labor presentation, pregnancy maintenance, pregnancy trimesters,* and *vitamin D.* The details of searched terms are provided in supplemental information.

### Eligibility criteria

#### Inclusion criteria

##### Types of study

Clinical trials with intervention and control groups.

##### Population

Pregnant women of any chronological age, gestational age and pregnancy complication with their infants.

##### Intervention

Vitamin D supplementation [D_2_ (ergocalciferol) or D_3_ (cholecalciferol)] either alone or in combination with a co-supplementation (intervention) with any form and supplementation duration.

##### Comparator(s)/control

Pregnant women who received placebo or no intervention or vitamin D at doses recommended by national guidelines for pregnant women.

##### Outcome

Changes in 25(OH)D, inflammatory markers including hs-CRP, TNF-α, transforming growth factor (TGF)-β, interferon (IFN)-γ, IL-1beta, IL-4, IL-6, IL-7, IL-8, IL-10, IL-13, and/or oxidative stress markers including MDA, TAC, GSH and superoxide dismutase (SOD)**.**

#### Exclusion criteria

1. Studies with observational, experimental/animal or in vitro design.
2. Studies with no random allocation or no comparing group.
3. RCTs had no measurement of circulating 25(OH)D and at least one of the inflammatory or oxidative stress biomarkers including hs-CRP, TNF-α, TGF-β, IFN-γ, IL-1β, IL-4, IL-6, IL-7, IL-8, IL-10, IL-13, and oxidative stress markers including MDA, TAC, GSH and SOD.
4. RCTs that implemented intervention in pregnant adolescents or in pregnancies occurred after in vitro fertilization (IVF) or in pregnant women with type 2 diabetes mellitus, asthma, autoimmune diseases, inflammatory bowel disease or renal diseases.
5. RCTs that assessed irrelevant outcomes, for instance, postpartum depression, airway functions, asthma in born, etc.

### Study selection

Firstly, the main sections including titles, abstracts and keywords of the retrieved studies by search strategy were read by two reviewers (SM_1_ & SM_2_). If the given information met the eligibility criteria the full text articles would be explored for more quality assessment in consultation with a second reviewer (RA). Decision making in the case of disagreement or uncertainty were resolved by discussion. If the two reviewers did not reach to an agreement by discussion, a third expert reviewer helped to reach consensus (BN). The articles not having met the inclusion criteria were excluded.

### Data extraction

A specific data extraction form, developed based on the study’s objectives and inclusion criteria, was used to extract the required data, including bibliographic (first author, journal name, volume and issue of journal, date of publication, date of execution), demographic (mother’s age, gestational age, occupation, race, socioeconomic level), anthropometric (weight, height, body mass index (BMI)), physical activity, methodologic data (sample size, study design, assay method), dose and duration of vitamin D supplementation and mean change values, standard deviations (SDs) and confidence intervals (CIs) of 25(OH)D, inflammatory and oxidative stress markers by two independent reviewers (SM_1_ & SM_2_). In terms of disagreement or uncertainty for data extraction, the reviewers discussed to reach an agreement, or a third expert reviewer (BN) would help in case the two reviewers could not reach to consensus. In case of missing required data, the reviewers contacted to the authors of the article, provided that their article would be used for the meta-analysis. During data synthesis, each intervention arm of the studies with more than one intervention group was considered as a single study.

### Risk of bias assessment

To evaluate the risk of bias in included RCTs, two investigators (SM_1_ and RA) reviewed the methods and quality of the included studies, separately. Then the studies were categorized according to the revised version (version 2) of “Cochrane Risk of Bias Assessment tool for randomized trials”, which has five main domains: randomization process, deviations from intended interventions, missing outcome data, measurement of the outcome and selection of the reported result. Each domain risk of bias judged to be “low risk of bias”, “some concerns” or “high risk of bias” according to the criteria presented in the Cochrane Handbook [16]. The criteria to make decision about overall risk of bias were as follow: 1) low risk of bias if all domains had low risk of bias for the result; 2) some concerns if there were some concerns in at least one domain for the result; 3) high risk of bias if there was high risk of bias in at least one domain for the result or there were some concerns for several domains in a way that diminished the reliability of the result [16]. If the two reviewers did not reach to an agreement by discussion, a third expert reviewer helped to reach a consensus (BN).

The quality of the evidence for each outcome was graded based on the Grading of Recommendations Assessment, Development and Evaluation (GRADE) approach [17]. It was assessed by two reviewers (SM_1_ & RA). The quality of evidence was classified as high, moderate, low, and very low [17].

### Data synthesis

A meta-analysis was done to evaluate the effect of vitamin D supplementation on inflammatory and/or oxidative stress markers. Due to the heterogeneity of studies, the random effect model (Der Simonian-Laird) method was used to pool the data and to estimate the Cohen’s standardized mean difference (SMD), weighted mean difference (WMD) and 95% CI. SMD was interpreted as trivial (0–0.19), low- (0.20–0.49), moderate- (0.50–0.79), and high-grade efficacy (≥ 0.80) [18]. The Cochran’s Q and I-squared (I^2^) tests were applied to assess statistical heterogeneity. I^2^ values of 0–24.9% indicated mild heterogeneity, 25–49.9% indicated moderate heterogeneity, 50–74.9% indicated high heterogeneity and ≥ 75% indicated severe heterogeneity [19].

### Sensitivity analysis

Sensitivity analysis was assessed by one by one study remove and calculation of the result without each specific study [20].

### Subgroup analysis

Subgroup analysis was used to detect the likely sources of heterogeneity. Studies were stratified according to the suspected variables assumed to be responsible for heterogeneity, including baseline vitamin D status according to the levels of 25(OH)D (deficient (< 50 nmol/L), insufficient (50–75 nmol/L) or sufficient (≥ 75 nmol/L)) [21], duration of vitamin D supplementation (< 15 weeks or > 15 weeks), dose of vitamin D supplementation (< 1000, 1000–2000, 2000–4000, > 4000 IU/d), vitamin D supplementation in control group (yes or no), having gestational complications such as preeclampsia or gestational diabetes mellitus (yes, no), gestational age at the beginning of study (< 12, 12–24, or > 24 weeks), receiving co-supplementation (yes, no) and also overall risk of bias of the RCTs (low risk of bias, some concerns and high risk of bias). Meta-regression was done in cases with less than 3 studies in a subgroup to adjust the effect of covariates on the results and to evaluate the relationship between pooled effect size and vitamin D dosage (IU /day).

### Publication bias

Possible publication bias was displayed by funnel plot and assessed by Egger’s method. To estimate the probable effect of missed studies on results, estimated studies were added to the funnel plot using ‘trim & fill method’ [22].

All statistical analyses were carried out using Stata 14.0 (Stata Corporation, TX, USA).

## Results

**Fig. 1:**
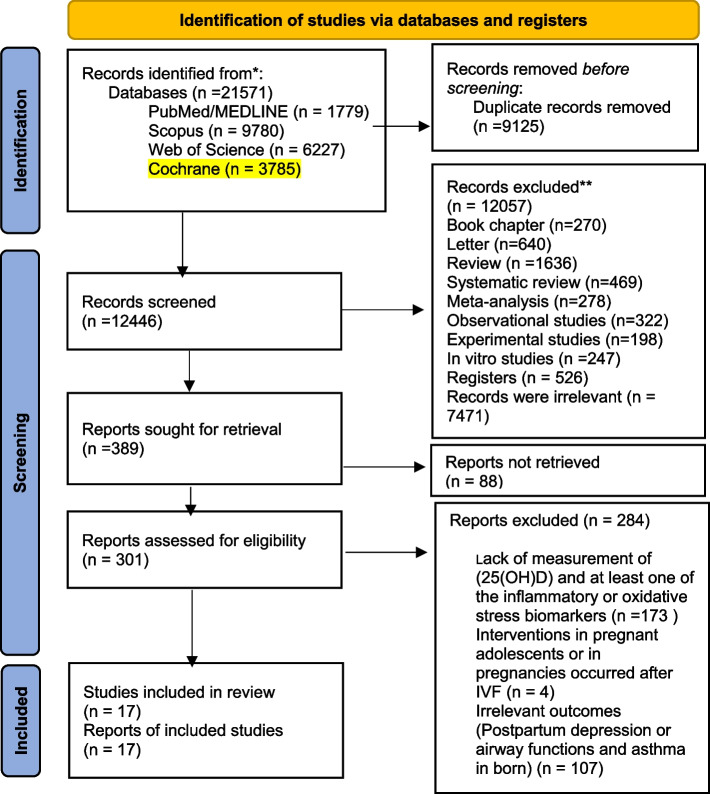
Vitamin D and inflammation and oxidative stress status in healthy or high risk pregnant women and their infants: a systematic review and meta-analysis

The process of data extraction and exclusion has been shown in Fig. 1. According to the imported syntax, 21571 records were identified from four databases (PubMed/MEDLINE: 1779, Scopus: 9780, WOS: 6227 and Cochrane: 3785). After removing duplicates (9125 studies), the remained articles were screened by title and abstract and subsequently 301 full text articles were evaluated for eligibility. Finally, 17 RCTs were included in the qualitative analysis and meta-analysis.

### Study characteristics

The detailed characteristics of 17 RCTs are presented in Table 1. Nine studies were conducted in Iran [10–12, 23–28], one in China [29], one in Bangladesh [30] three in the USA [31–33], one in the UK [34], one in Brazil [35] and one in India [36]. The age range of participants was 18–40 years and gestational age at the first visit was 8 to 32 weeks. Duration of supplementation was between 6 and 29 weeks. We considered each intervention arm of the studies with more than one intervention group as a single study. For example, the studies by Razavi [11] et al. and Zhang et al. [29] were considered as two and three studies, respectively. The included RCTs totally comprised 1,465 participants (776 in intervention and 689 in control groups). Total number of cord blood specimens was 162 (84 in intervention and 78 in control groups).

**Table 1:** Summary of the randomized clinical trials (RCTs)

**First author, year**	**Design**	**Participants, n**		**Place**	**Health condition**	**Gestational age (wk)**	**Duration (wk)**	**Treatment**	**Control**	**Explored variables**	**Outcome**
		**treatment**	**control**								
Rodrigues Amorim Adegboye, 2021 [35]	RCT	13	9	Rio de Janeiro, Brazil	Healthy	16.2 ± 2.4	< 20 to 30–38 wk	fortified sachet with vitamin D (500 IU) and calcium (500 mg) twice a day	placebo sachet	25(OH)D, CRP	CRP levels had no significant differences between supplemented and placebo groups
Khatiwada, 2021 [9, 33]	RCT	110	107	South Carolina, USA	Healthy	10–14	> 20	4400 IU vitamin D3/day	400 vitamin D3/day	25(OH)D, TGF-β, IFN-γ, CRP, IL-2, IL-4, IL-5, IL-10, VEGF,	Immune-mediators in the late pregnancy did not change in response to vitamin D supplementation during pregnancy
Gunasegaran, 2021 [36]	RCT	34	36	Tamil Nadu, India	GDM	24–28	6	vitamin D 1000 IU and calcium 1000 mg	itamin D 250 IU and calcium 500 mg	25(OH)D, GSH	Supplementation with 1000 IU vitamin D and 1000 mg Calcium, had a positive effect on oxidative stress in women with GDM
Motamed, 2020 [23]	RCT	37	37	Tehran, Iran	Healthy	8–12	28.7	2000 IU/d vitamin D3 + current supplementation during pregnancy	1000 IU/d vitamin D3 + current supplementation during pregnancy	MDA, TAC in the serum of mothers and offsprings’ cord blood	No significant within & between group differences in serum and cord blood concentration of MDA and TAC
Motamed, 2019 [24, 37]	RCT	37	37	Tehran, Iran	Healthy	8–12	28.7	2000 IU/d vitamin D3 + current supplementation during pregnancy^a^	1000 IU/d vitamin D3 + current supplementation during pregnancy	25(OH)D, hs-CRP, and cell-culture supernatant concentrations of IL-1 beta, IL-6, and TNF-α in mothers and offsprings’ cord blood	A significant decrease of TNF-α in maternal PBMCs of 2000 IU/d vitamin D group & lower concentration of cord blood IL-6 in 2000 IU/d compared to 1000 IU/d vit D group
Jamilian, 2019 [10]	RCT	30	30	Kashan, Iran	Healthy	24–28	6	200 IU/d vitamin D3 + 100 mg/d magnesium + 4 mg/d zinc + 400 mg calcium/d	Placebo	25(OH)D, hs-CRP, MDA, TAC in the maternal serum	Intervention caused a significant decrease in serum hs-CRP & plasma MDA & an increment in TAC levels compared to placebo group
Braithwaite, 2019 [34, 39]	RCT	93	102	UK	Healthy	10–17	20.5	1000 IU/d vitamin D3	Placebo	25(OH)D, CRP in the maternal plasma	Vitamin D 3 supplementation had no effect on CRP status
Hornsby, 2018 [32]	RCT	26	25	Boston, USA	Healthy	10–18	25	4400 IU/d vitamin D3	400 IU/d vitamin D3	25(OH)D, IFN- γ, IL-1 β, IL-6, and IL-8 in the supernant of cultured CBMCs	The levels of IFN-γ, IL-1β, IL-6, and IL-8 in CBMCs of 4400 IU/d vitamin D group increased
Razavi, 2017 [11, 38]	RCT	30	30	Tabriz, Iran	GDM	24–28	6	T1: 50,000 IU vitamin D3 every 2 week T2: 50,000 IU vitamin D3 every 2 week + 1000 mg/d omega-3	Placebo	serum levels of 25(OH)D, hs_CRP and plasma concentration of MDA, TAC, GSH in mothers	vitamin D + Omega3 (T1) decreased the concentration of hs-CRP, MDA & increased TAC & GSH compared to other groups
Yazdchi, 2016 [25]	RCT	38	38	Tabriz, Iran	GDM	24–28	8	50,000 IU twice a month vitamin D3	Placebo	25(OH)D, hs-CRP in the maternal serum	A significant increment of hs-CRP in placebo group, but no significant change in intervention group
Akhtar, 2016 [30]	RCT	80	80	Bangladesh	Healthy	26–29	16.5	35,000 IU/wk vitamin D3	Placebo	25(OH)D, IL-10 TNF-alpha IFN- γ in the cultured CBMCs	higher concentrations of IL-10 & TNF-α & IFN-γ in the vitamin D group compared to the placebo
Samimi, 2016 [12]	RCT	30	30	Kashan, Iran	at risk for pre-eclampsia	20–32	12	50 000 IU vitamin D3 every 2 weeks + 1000 mg/ d calcium	Placebo	GSH in the maternal plasma	Plasma concentrations of GSH increased compared to placebo
Zhang, 2016 [29]	RCT	38 38 37	20	Shanghai, China	GDM	24–28	From 24–28 wk of gestation until delivery	T1: 200 IU/d vitamin D3 T2: 2000 IU/d vitamin D3 T3: 4000 IU/d vitamin D3	Control	25(OH)D in the serum and TAC and GSH in the plasma of mothers	TAC & GSH levels increased in response to T3 (50,000 IU every 2 weeks (4,000 IU daily for 12.5 days)) compared to other groups
Zerofsky, 2016 [31, 40]	RCT	26	29	California, USA	Healthy	20	22	2000 IU/d vitamin D3	400 IU/d vitamin D3	25(OH)D, IL-10 in the maternal plasma	2000-IU/d resulted in a significant increase in the percentage of CD4 + IL-10 + T cells compared to 400-IU/d that showed a 12% decrease in the same biomarker from the first to third visit
Asemi, 2014 [26]	RCT	28	28	Kashan, Iran	GDM	24–28	6	1,000 mg/d Calcium + 50,000 IU vitamin D3 twice a month	Placebo	25(OH)D in the serum and hs-CRP, MDA, GSH, TAC, NO in the plasma of mothers	Intervention caused a significant increase in GSH and prevented the increase of MDA levels compared to the placebo
Asemi, 2013 (a) [27]	RCT	27	27	Kashan, Iran	GDM	24–28	6	50,000 IU vitamin D3 twice a month	Placebo	25(OH)D, hs-CRP in the maternal serum	hs-CRP had a significant decrease in vitamin D group compared to the placebo group
Asemi, 2013 (b) [28]	RCT	24	24	Kashan, Iran	Healthy	25	9	400 IU/d vitamin D3	Placebo	25(OH)D, calcium, hs-CRP in the serum MDA & GSH in the plasma of mothers	A significant decrease in serum hs-CRP in intervention group

*n* Number, *wk* Week, *GDM* Gestational diabetes, *25(OH)D* 25*-​*hydroxyvitamin D*3*, *hs-CRP* High-sensitivity C-reactive protein, *IL* Interleukin, *TNF-α* Tumor Necrosis Factor-alpha, *MDA* Malondialdehyde, *TAC* Total antioxidant capacity, *IFN-γ* Interferon Gamma, *GSH* Glutathione, *NO* Nitric oxide, *CRP* C-reactive protein, *TGF-β* Transforming growth factor-beta, *CBMCs* Cord blood mononuclear cells

^a^Current supplementation during pregnancy: daily iron, folic acid and multivitamin supplementation

Overall risk-of-bias judgment: Low risk of bias: The study is judged to be at low risk of bias for all domains for this result; Some concerns: The study is judged to raise some concerns in at least one domain for this result, but not to be at high risk of bias for any domain; High risk of bias: The study is judged to be at high risk of bias in at least one domain for this result, or the study is judged to have some concerns for multiple domains in a way that substantially lowers confidence in the result

### The effect of vitamin D supplementation on changes in circulating 25(OH)D concentration

Of 17 RCTs, one study had measured circulating 25(OH)D concentration just at the baseline and in the cord blood [30]. Besides, in two studies the report of 25(OH)D concentration had been duplicated by the same author [23]. Therefore, the data of one of them was included in the meta-analysis.

**Fig. 2:**
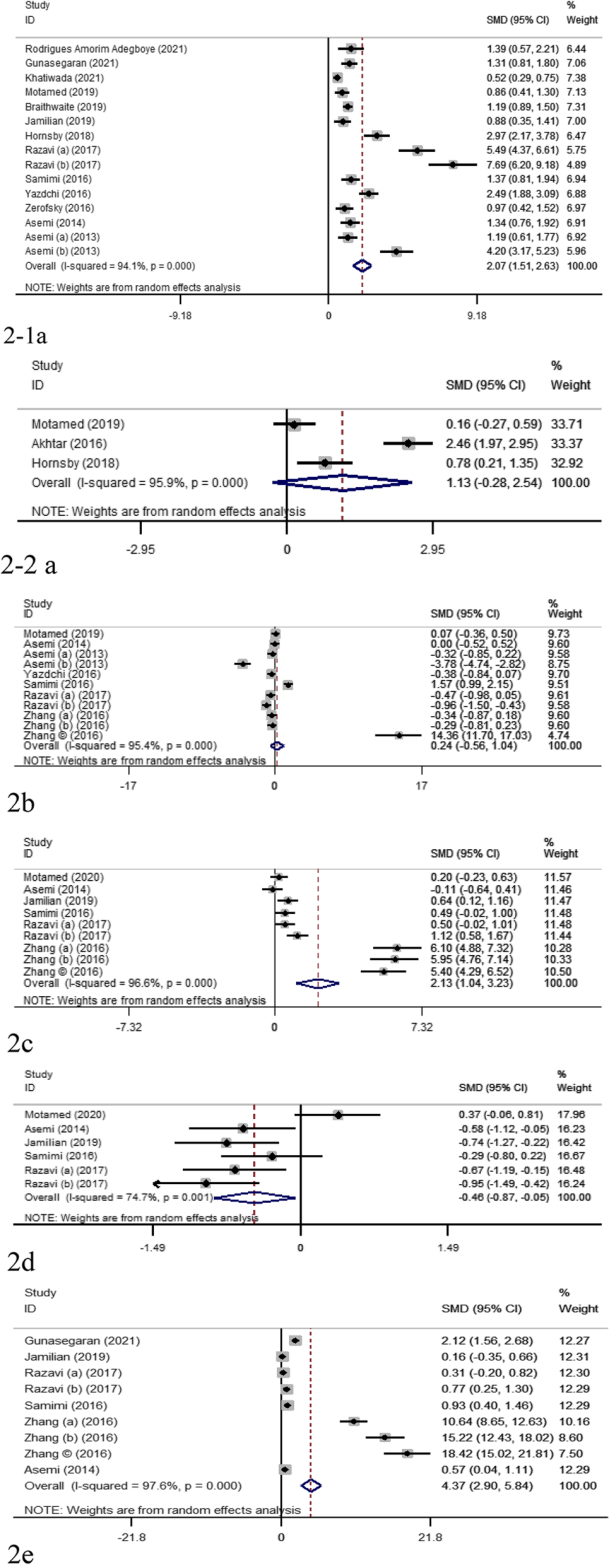
Forest plot and SMD with 95% CI estimate the effect of vitamin D supplementation on the concentration of 25(OH)D (1a; maternal, 1-1a cord blood), hs-CRP (1b), TAC (1c), MDA (1d) and GSH (1e). The square shapes represent weight of the articles in the analysis and diamond reflects the pooled SMD

The results of the random-effect model showed that vitamin D supplementation significantly increased 25(OH)D concentrations (SMD: 2.38; 95% CI 1.67, 3.08). However, there was severe heterogeneity among included studies (I^2^ = 94.1%, *p* < 0.001) (Fig. 2-1a). After sensitivity analysis, the pooled effect of vitamin D supplementation on 25(OH)D concentration remained the same (SMD 2.38; 95% CI 1.68, 3.08). Pooled WMD also showed an increase of 31.36 nmol/L (95% CI 21.31, 41.02) in circulating 25(OH)D concentration after vitamin D supplementation. Sub-group analysis showed that duration and dose of supplementation, and gestational age were the possible sources of heterogeneity in RCTs as a significant change in I^2^ was observed in the subgroup of women with 1000–2000 IU/d vitamin D supplementation (k = 3, *n* = 384, I^2^ = 0.0%, *P* = 0.50) (Supplementary Table 1). Based on the results of meta-regression, none of the variables explained the existed heterogeneity (supplementary Table 6). Funnel plot and Egger’s test have depicted the possibility of publication bias (k = 12, B = 8.98, t = 4.69, *P* = 0.001) (Table 2). Applying Trim and fill method, one study was added to correct the publication bias but the value of effect size showed no significant change.

**Table 2:** Publication bias assessment by Egger’s statistical test for effect of vitamin D supplementation on the levels of 25(OH)D, CB 25(OH)D, hs-CRP, MDA, TAC and GSH

**Markers**	**NO of interventions**	**NO of participants**	**Coefficient**	**Std. Err**	**t**	***P***	**95% CI**	
							**LL**	**UL**
25(OH)D	15	1061	7.56	1.33	5.65	< 0.001	4.67	10.45
CB 25(OH)D	3	162	12.06	32.81	0.37	0.77	-404.05	429.05
hs-CRP	11	670	6.05	4.63	1.31	0.22	-4.42	16.53
TAC	9	543	15.35	1.32	11.63	< 0.001	12.23	18.47
MDA	6	370	-21.99	3.32	6.62	0.003	-31.21	-12.77
GSH	9	539	12.73	1.30	9.78	< 0.001	9.65	15.81

*Abbreviations*: *25(OH)D* 25*-​*hydroxyvitamin D*3*, *CB* Cord Blood, *CI* Confidence interval, *hs-CRP* High-sensitivity C-reactive protein, *TAC* Total antioxidant capacity, *MDA* Malondialdehyde, *GSH* Glutathione

Three studies measured the concentration of 25(OH)D in cord blood [24, 30, 32]. According to the meta-analysis vitamin D supplementation had no significant effect on cord blood concentration of 25(OH)D (SMD: 1.13; 95% CI -0.27, 2.54). Nevertheless, there was severe heterogeneity among included studies (I^2^ = 95.9%, *p* < 0.001) (Fig. 2–2a). According to the meta-regression, none of the confounding factors explained the observed heterogeneity (supplementary Table 6). According to egger’s test (Table 2) and funnel plot, a significant publication bias was observed. However, trim and fill did not add any studies.

### The effect of vitamin D Supplementation on hs-CRP

Serum hs-CRP has been measured in 11 RCTs (Table 1). According to the results of the applied model, among heterogeneous studies, vitamin D supplementation in pregnant women had no significant effect on hs-CRP concentrations (SMD: 0.27; 95% CI -0.52, 1.06; I^2^ = 95.4%) (Fig. 2b). Sensitivity analysis showed that the pooled SMD regarding the effect of vitamin D supplementation on hs-CRP remained almost constant after removing each study.

Based on subgroup analysis, dose of supplementation could be a potential source of heterogeneity, as I^2^ decreased to 11.7% (SMD: -0.07; 95% CI -0.43, 0.27) in the sub-group of 1000–2000 IU/d vitamin D (Supplementary Table 2); however, the number of studies was insufficient for a conclusive result (k = 2, *n* = 132). Consequently, meta-regression was done but none of the suspected variables explained the existed heterogeneity (Supplementary Table 6).

According to the Egger’s test (Table 2) and funnel plot, there was low possibility of publication bias for meta-analyses evaluating the impacts of vitamin D supplementation on hs-CRP (B = 6.05, t = 1.31, *p* = 0.22). Trim and fill method added no studies which confirms low risk of publication bias.

### The effect of vitamin D Supplementation on changes of cytokines

A number of cytokines including TNF-α (k = 2), IFN-γ (k = 3), IL-1 (k = 2), IL-6 (k = 2), and IL-10 (k = 3) were examined by the included articles (Table 1). However, because of insufficient number of studies we were unable to do meta-analysis for these cytokines.

Khatiwada et al. found that immune-mediators (TGF-β, IFN-γ, IL-2, IL-4, IL-5, IL-10 and VEGF) in the late pregnancy were not affected by vitamin D supplementation during pregnancy [33].

Motamed et al. 2019 measured cytokines (IL-1β, IL-6 and TNF-α) in cultured peripheral blood mononuclear cells (PBMCs) of mothers and cord blood serum and showed a significant decrease in TNF-α with 2000 IU/d vitamin D supplementation, but no change in other cytokines. However, cord blood serum concentration of IL-6 in 2000 IU/d was significantly lower than 1000 IU/d vitamin D group [24]. In the study of Akhtar et al. 2016 who evaluated the effect of vitamin D supplementation on cultured cord blood mononuclear cells (CBMCs) of participants, higher concentrations of IL-10 and TNF-α in the vitamin D group after iCD3/iCD28 stimulation and IFN-γ with phytohemagglutinin (PHA) stimulation was observed compared with the placebo [30]. Hornsby et al. 2018 found an enhance in the levels of proinflammatory cytokines including granulocyte macrophage colony stimulating factor (GMCSF), IFN-γ, IL-1b, IL-6, and IL-8 in CBMCs of mothers supplemented with 4400 IU/d vitamin D3 [32]. In the study of Zerofsky et al. (2016), vitamin D supplementation with 2000 IU/d resulted in significantly increased percentage of CD4 + IL-10 + T cells compared with 400 IU/d that showed a 12% decrease in the same biomarker from the first to third visit [31].

### The effect of vitamin D Supplementation on changes of oxidative stress biomarkers

A number of oxidative stress biomarkers such as TAC (k = 6), MDA (k = 5), GSH (k = 5), and nitric oxide (k = 1) were measured by the included RCTs.

A total of 6 RCTs examined the effects of vitamin D supplementation on TAC [10–12, 23, 26, 29]. The heterogeneity was significant (I^2^ = 96.6%, *p* < 0.001). A random-effect model showed a significant increase in TAC in response to vitamin D supplementation (SMD 2.13; 95% CI 1.04, 3.23) (Fig. 2c). TAC was increased after vitamin D supplementation (WMD: 63.66 mmol/L; 95% CI 21.75, 105.50).

Sensitivity analysis confirmed that the result was not sensitive to one study. Also, sub-group analysis for TAC showed mild heterogeneity in more than 15 weeks of vitamin D supplementation group (k = 2, *n* = 134; SMD: 0.32, I^2^ = 0.0%; *p* = 0.39) and in healthy mothers (k = 3, *n* = 194; SMD: 0.41; I^2^ = 0.0%; *p* = 0.41) (Supplementary Table 3). In terms of overall risk of bias, studies in the “some concerns” category, could be considered as potential sources of heterogeneity (I^2^ = 15.8%, *p* = 0.31) (Supplementary Table 3). The funnel plots depicted for publication bias for TAC and Egger’s test confirmed this notion (B = 15.35, t = 11.63, *p* < 0.001) (Table 2). The trim and fill method added one study for correcting the publication bias and estimated SMD changed to 1.52, 95%CI, 0.34, 2.69.

To evaluate the effect of vitamin D supplementation on the concentration of MDA, six studies [10–12, 23, 26] were included in meta-analysis. The observed heterogeneity was severe (*p* = 0.001, I^2^ = 74.71%).

As shown in Fig. 2d, vitamin D supplementation resulted in a significant decrease in serum MDA concentration (SMD: -0.46; 95% CI -0.87, -0.05) up to an average of -0.69 μmol/L (95% CI -0.98, -0.39). Sensitivity analysis showed the pooled result was not sensitive to any specific study. The results of sub-group analysis showed a negligible heterogeneity in studies lasted < 15 weeks (k = 4, *n* = 134, I^2^ = 0.0%, *p* = 0.80), those with 2000–4000 IU/d vitamin D supplementation (k = 4, *n* = 236, I^2^ = 5.4%, *p* = 0.36), the control groups with no supplementation of vitamin D (k = 5, *n* = 266, I^2^ = 0.0%, *p* = 0.50), and studies with pregnancy complications (k = 5, *n* = 236, I^2^ = 0.0%, *p* = 0.50) (Supplementary Table 4). Moreover, co-supplementation was another source of heterogeneity of the pooled result, as the result was more consistent in studies with co-supplementation (k = 4, *n* = 236, SMD: -0.63; I^2^ = 10.4%, *p* = 0.34) (Supplementary Table 4). When it came to the overall risk of bias, the results were robust in “some concerns” subgroup (SMD: -0.65; I^2^ = 9.3%, *p* = 0.34) (Supplementary Table 4). Through meta-regression, we found that duration of supplementation (B = 0.05, *p* = 0.01) and sample size (B = -0.06, *p* = 0.02) were responsible for the observed heterogeneity between studies (Supplementary Table 6). Egger’s test (B = -21.99, t = 6.62, *P* = 0.003) (Table 2) and funnel plot showed a possibility of publication bias, but trim and fill correction added no further studies. Therefore, publication bias in this regard could not be the case of concern.

Six studies including nine interventions were included to evaluate the effect of vitamin D on circulating GSH concentration [10–12, 26, 29]. The meta-analysis showed a severe heterogeneity among the studies (SMD: 4.80; 95%CI 3.13, 6.47; I^2^ = 97.7%, *p* < 0.001, Fig. 2e). The concentration of GSH increased significantly in response to vitamin D supplementation (WMD: 139.39; 95% CI 98.63, 180.16). Sensitivity analysis showed the result was only sensitive to the study by Zhang et al. 2016 [29]. Based on subgroup analysis, the result was robust for taking more than 2000–4000 IU/d vitamin D (k = 4, *n* = 236, SMD:0.63; I^2^ = 1.91%, *p* = 0.38), co-supplementation (k = 4, *n* = 233, SMD:0.60; I^2^ = 37.9%, *p* = 0.18), and subgroup with “some concerns” regarding risk of bias (SMD:0.53; I^2^ = 48.1%, *p* = 0.12) (supplementary Table 6). According to the egger’s test, publication bias was possible (B = 12.76, t = 21.33, *p* < 0.001) (Table 2). Trim and fill method added one study to correct the publication bias, and the added study changed the SMD from 3.10 (95% CI 1.28, 4.92) to 4.80 (95% CI, 3.13, 6.47). Nevertheless, before and after adding one study, the effect size was in the strong range.

### Risk of bias assessment of RCTs

**Fig. 3:**
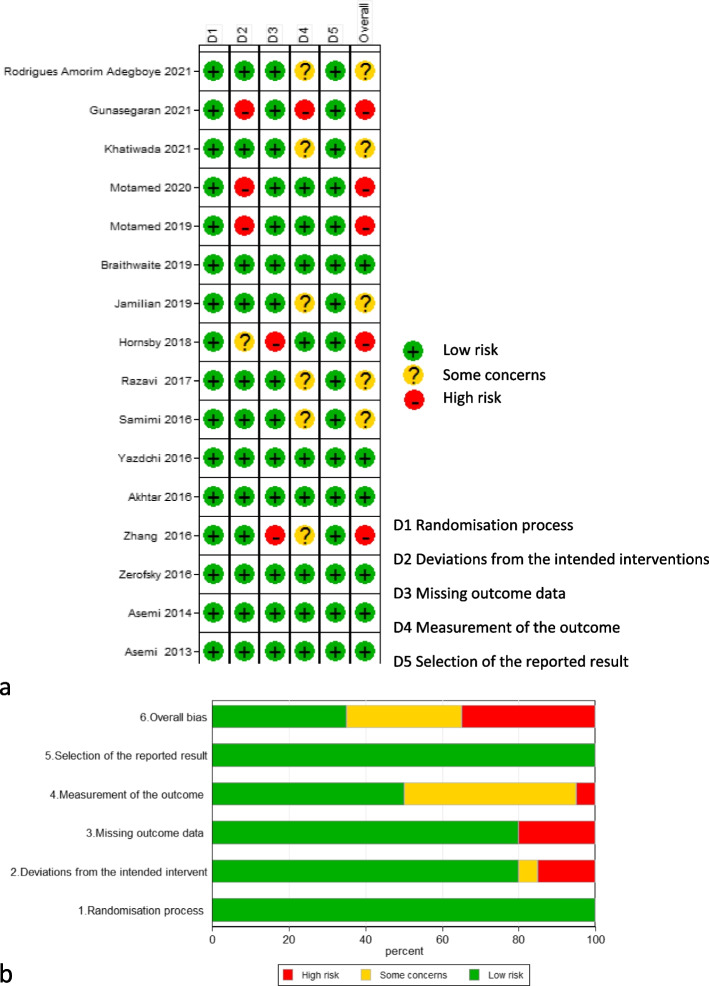
Risk of bias assessments for studies evaluating the effect of vitamin D supplementation on the level of 25(OH)D, hs-CRP, TAC, MDA and GSH (**a**) and the percentages of low risk of bias (green), some concerns (yellow) and high risk of bias (red) for each domain of Cochrane risk of bias assessment tool (**b**)

The result of risk of bias assessment has been depicted in Fig. 3 (a & b). As it has been shown in Fig. 3a, the overall risk of bias of studies was as follow: five studies judged to have high risk of bias [23, 29, 32, 36, 37], there were some concerns regarding five studies [10, 12, 33, 35, 38], and six studies had low risk of bias [25–27, 30, 39, 40]. Figure 3b shows the percentages of low risk of bias (green), some concerns (yellow) and high risk of bia (red) for each domain of Cochrane risk of bias assessment tool.

### GRADE assessment of RCTs

The result of GRADE assessment is presented in Table 3. Accordingly, the certainty of evidence for maternal 25(OH)D, TAC, MDA and GSH was “low” and it was shown to be “very low” for hs-CRP and 25(OH)D in the cord blood.

**Table 3:** GRADE assessment of the certainty of evidence regarding the effect of vitamin D supplementation on inflammatory and oxidative stress markers

**Biomarker**	**No of interventions**	**Risk of bias1**	**Inconsistency (heterogeneity)2**	**Indirectness3**	**Imprecision4**	**Publication bias 5**	**Effect size6**	**Certainty of evidence**
25(OH)D	15	Very serious	Very serious	Serious	Not serious	Serious	+ 2	Low
CB 25(OH)D	3	Very serious	Very serious	Serious	Very serious	Very serious	+ 2	Very low
hs-CRP	11	Very serious	Very serious	Serious	Serious	Not serious	0	Very low
TAC	9	Very serious	Very serious	Serious	Not serious	Serious	+ 2	Low
MDA	6	Very serious	Serious	Serious	Not serious	Serious	0	Low
GSH	9	Very serious	Very serious	Serious	Not serious	Serious	+ 2	Low

1 risk of bias: no problem = not serious (0); problem with 1 element = severe (-1); problem with 2 or more elements = very severe (-2)

2 inconsistency: I2 < 50% = not serious (0), 50–75% = serious (-1), > 75% = very serious (-2)

3 indirectness or variation in participants, intervention, outcome variables: no problem = low; problem with 1 element = serious (-1); problem with 2 or more elements = very serious (-2)

4 imprecision: > 5 studies = not serious (0); 4 to 5 studies = serious (-1); 3 or less studies = very serious (-2)

5 publication bias: no publication bias = not serious, publication bias proved by only one method of assessment = serious, publication proved by more than one method of assessment besides a considerable difference between the combined and pure measurement = very serious

6 scoring: lower than 0.5 = 0, between 0.5 and 0.79 =  + 1, 0.8 or higher =  + 2

*Abbreviations*: *25(OH)D* 25-​hydroxyvitamin D3, *CB* Cord Blood, *hs-CRP* High-sensitivity C-reactive protein, *K* Number of studies, *TAC* Total antioxidant capacity, *MDA* Malondialdehyde, *GSH* Glutathione

## Discussion

In the present study we evaluated the effect of vitamin D supplementation on changes in 25(OH)D and selected circulating inflammatory and oxidative stress biomarker levels which have been reported by individual RCTs in pregnant women.

It is worth to mention that due to scarcity of studies addressing our intended outcomes, and also remarkable diversity across the existing reports, conducting a meta-analysis was possible only for some data of RCTs including 25(OH)D, hs-CRP, TAC, MDA and GSH.

### Calcidiol (25(OH)D)

In the present study, the result of meta-analysis of thirteen studies showed a significant effect of vitamin D supplementation on the increase of maternal circulating 25(OH)D concentration. Although, the effect size was strong, the certainty of evidence was low due to the fact that risk of bias and heterogeneity were very serious and also indirectness and publication bias were serious. Consistently, other systematic reviews including the one that involved 13 RCTs published from 2000 to 2018 [41] and that with 13 RCTs published between 1980 and 2014 [42] showed the same result and similar severity of heterogeneity. It can be argued that in the present meta-analysis only those studies that had examined 25(OH)D concentration alongside at least one of the inflammation or oxidative stress biomarkers were included. Consequently, the observed heterogeneity can be attributed to the limited number of included studies.

### hs-CRP

According to the meta-analysis of merged data, vitamin D supplementation had no effect on the concentration of hs-CRP. GRADE assessment showed that the certainty of evidence in this regard was “very low” as a result of very serious risk of bias and heterogeneity, as well as serious indirectness and imprecision and also weak effect size. On the contrary, the meta-analysis of 10 trials with a total of 924 participants indicated a significant decrease in hs-CRP level after vitamin D supplementation [43]. Another meta-analysis among women with polycystic ovary syndrome (PCOS) confirmed the positive effect of vitamin D on reducing serum hs-CRP [44]. The observed controversy between the results of the present study and other studies may be as a consequence of different population groups and different number of included articles in the meta-analyses. It is noteworthy that due to very low certainty of the current evidence according to GRADE approach, the result of the effect of vitamin D supplementation on the level of hs-CRP has to be reported conservatively and the replication of evaluating the same outcomes in larger data is warranted.

### Cytokines

Original articles included in this review had measured various cytokines in different environments (cell culture, maternal serum and /or cord blood samples) in different stages of pregnancy. Therefore, due to lack of sufficient data on cytokines, the results of single studies are discussed in this section.

In the study of Motamed et al., the level of cytokines (IL-1β, IL-6 and TNF-α) were assayed in cultured PBMCs of mothers and cord blood serum. Following supplementation with 2000 IU/d vitamin D from the first trimester, a significant decrease of TNF-α in cultured PBMCs and IL-6 in cord blood serum concentration was observed [24]. On the contrary, in another study after vitamin D supplementation, higher concentrations of IL-10, TNF-α and IFN-γ were found compared with the placebo [30]. Similarly, CBMCs levels of proinflammatory cytokines including IFN-γ, IL-1β, IL-6, and IL-8 were enhanced in those subjects supplemented with 4400 IU/d vitamin D compared with the control group in the study of Hornnsby et al. [32]. McManus et al., through conducting a case–control study suggested a positive relationship between maternal serum concentration of 25(OH)D and IL-8 and TNF-α in GDM cases [45]. A positive correlation between 25(OH)D and TNF-α was also detected in pregnant women with hypertensive disorders [46]. Nonetheless, Mousa et al., found a significant adverse correlation between maternal concentrations of 25(OH)D and IL-6 at 12–15 week of gestation among overweight or obese pregnant women who were prone to GDM [47]. In another cross-sectional study launched by Haidari et al., in 45 GDM and 45 healthy pregnant women, an adverse correlation between the concentration of 25(OH) D and hs-CRP in the serum of healthy subjects was found [48]. Obviously there is a large amount of controversies among the available evidence regarding the effectiveness of vitamin D supplementation on cytokines or the association between circulating 25(OH)D and cytokine concentrations. Some of these results contradicted some other studies among non-pregnant women which have documented that vitamin D might influence inflammation through down-regulating IL-6 and TNF-α [49–51].

Progesterone-induced blocking factor (PIBF), a protein produced by progesterone-stimulated lymphocytes, has immunomodulatory effects on CD4 + T cells probably through membrane progesterone receptors (mPRs) [52]. It has recently been found that vitamin D may upregulate PIBF in activated human peripheral lymphocytes and it is likely that vitamin D and progesterone exert their anti-inflammatory including IL-6-suppressing effects synergistically through PIBF [53].

### Oxidative stress biomarkers

Based on our meta-analysis, vitamin D supplementation decreased serum MDA and increased TAC and GSH levels. However, the certainty of these findings was low when all items of GRADE assessment including risk of bias, heterogeneity, indirectness, imprecision, publication bias and also the strength of effect size were considered together. Therefore, these results have to be interpreted with caution. The results of some other systematic reviews also revealed positive effect of vitamin D supplementation on the levels of the same biomarkers among women with PCOS [44] and diabetic patients [54]. However, there are some evidence that does not confirm this effect [55]. The results of the studies that implemented less than 100,000 IU Vitamin D per month found no impact of vitamin D on the levels of GSH and TAC. Nevertheless, treatment with doses higher than 100,000 IU per month resulted in an increased level of GSH [56]. The results of the same study have shown that MDA level is more responsive to vitamin D supplementation using high dose biweekly as compared with smaller doses in daily or weekly basis. Furthermore, the most effectiveness of doses between 100,000 and 200,000 IU per month on decreasing the level of MDA was detected [56]. The evidence demonstrated that vitamin D reduces the production of free radicals by interfering the NF-κB-dependent pathways [57, 58], and decreasing lipid hydrogen peroxide at the cellular membrane [59]. Suppression of the expression of nicotinamide adenine dinucleotide phosphate (NADP) enzyme [60], and restraining the aggregation of the advanced glycation end products [61], are another mechanisms by which vitamin D may suppress oxidative stress status.

### Strengths and limitations

To the best of our knowledge, this is the first systematic review and meta-analysis on the effects of vitamin D supplementation on inflammatory and oxidative stress biomarkers during pregnancy. In the present study the principles of PRISMA method for conducting a systematic review including comprehensive search strategy and quality assessment were followed. We also employed GRADE approach to assess the certainty of evidence by considering items that might influence the observed results.

There are also a few limitations in the present study, including limited number of included articles and small sample size of each of them. Furthermore, various doses and duration of vitamin D supplementation and also co-supplementation of calcium, omega-3 and other micronutrients in some studies, made it difficult to distinguish the efficacy of vitamin D on the biomarkers. Moreover, because of statistical limitations and limited number of studies we were unable to do meta-analysis for all markers.

## Conclusion

Our meta-analysis showed increased levels of 25(OH)D, TAC and GSH and a reduction in the levels of MDA but no changes of hs-CRP in response to vitamin D supplementation alone or in combination with other nutrients during pregnancy. These results were somehow compatible with the findings of single studies. However, these evidence are stated with caution due to low or very low certainty.

## Supplementary Information

**Additional file 1.**

## Data Availability

The datasets of the present study are available from the corresponding author on reasonable request.

## Abbreviations

D3: Cholecalciferol
D2: Ergocalciferol
GSH: Glutathione
IFN-γ: Interferon γ
MDA: Malondialdehyde
RCTs: Randomized Controlled Trials
SOD: Superoxide dismutase
TAC: Total antioxidant capacity
TGF-beta: Tumour growth factor
