# Supplementary material for: The effect of vitamin D supplementation on oxidative stress and inflammatory biomarkers in pregnant women: a systematic review and meta-analysis of clinical trials
Source: BMC Pregnancy Childbirth. 2022 Nov 5;22:816. doi: 10.1186/s12884-022-05132-w (PMC9636796; doi:10.1186/s12884-022-05132-w)
Supplement: Supplementary file 1 — Additional file 1. [file 12884_2022_5132_MOESM1_ESM.docx]

Supplemental data

**Title: The effect of vitamin D supplementation on oxidative stress and inflammatory biomarkers in pregnant women: a systematic review and meta-analysis of clinical trials**

Soudabe Motamed^1^, Bahareh Nikooyeh^2^, Razieh Anari^2^, Somayeh Motamed^3^, Zeinab Mokhtari^4^, Tirang Neyestani^2^

**The search terms for PubMed was as follow:**

(pregnan*[tiab] OR gestation[tiab] OR “child bearing”[tiab] OR childbearing[tiab] OR gravidity[tiab] OR “intrauterine pregnancy”[tiab] OR “labor presentation”[tiab] OR “labour presentation”[tiab] OR “pregnancy maintenance”[tiab] OR “pregnancy trimesters”[tiab]) AND (“vitamin d”[tiab] OR 25(OH)D[tiab] OR “25(OH) D”[tiab] OR “25 (OH) D”[tiab] OR “25 hydroxy vitamin D 3”[tiab] OR 25(OH)D3[tiab] OR “25(OH) D3”[tiab] OR “25 (OH) D3”[tiab] OR “1, 25 dihydroxy vitamin D 3”[tiab] OR calcifediol[tiab] OR calcitriol[tiab] OR Calciol[tiab] OR “Vitamin D 3”[tiab] OR “Vitamin D3”[tiab] OR Cholecalciferol*[tiab] OR “Hydroxyvitamins D”[tiab] OR Hydroxycholecalciferol[tiab] OR Calciferol*[tiab] OR “Vitamin D 2”[tiab] OR “Vitamin D2”[tiab] OR Ergocalciferol[tiab] OR “25 Hydroxyvitamin D 2”[tiab] OR “25 Hydroxyergocalciferol”[tiab] OR “25-Hydroxyvitamin D2”[tiab] OR “25 Hydroxyvitamin D2”[tiab] OR 25-Hydroxycalciferol[tiab] OR “25 Hydroxycalciferol”[tiab] OR “(3 beta,5Z,7E)-9,10-Secocholesta-5,7,10(19)-trien-3-ol”[tiab] OR Calciferols[tiab] OR “25-hydroxyvitamin D”[tiab] OR “24,25 dihydroxyvitamin D”[tiab] OR “25 hydroxyvitamin D”[tiab] OR “9,10 secocholesta 5,7,10(19) trien 23 yne 1,3,25 triol”[tiab] OR “9,10 secocholesta 5,7,10(19) trien 23 yne 3,25 diol”[tiab] OR “9,10 secocholesta 5,7,10(19),16 tetraen 23 yne 1,3,25 triol”[tiab] OR “9,10 secocholesta 5,7,10(19),22 tetraene 1,3,25,26 tetrol”[tiab] OR “ascorbic acid plus fluoride plus retinol plus vitamin D”[tiab] OR “calcium carbonate plus ferrous fumarate plus vitamin D”[tiab] OR “calcium phosphate dibasic plus ferrous sulfate plus manganese sulfate plus nicotinic acid plus riboflavin plus thiamine plus vitamin D”[tiab] OR “9,10-Secoergosta-5,7,10(19),22-tetraene-3 beta,25-diol”[tiab] OR Ercalcidiol[tiab] OR Tachystin[tiab] OR Dihydrotachysterin[tiab] OR Calcamine[tiab])

**The search terms for web of science was as follow:**

(TS=(pregnan*) OR TS=(gestation) OR TS=(“child bearing”) OR TS=(childbearing) OR TS=(gravidity) OR TS=(“intrauterine pregnancy”) OR TS=(“labor presentation”) OR TS=(“labour presentation”) OR TS=(“pregnancy maintenance”) OR TS=(“pregnancy trimesters”)) AND (TS=(“vitamin d”) OR TS=(25(OH)D) OR TS=(“25(OH) D”) OR TS=(“25 (OH) D”) OR TS=(“25 hydroxy vitamin D 3”) OR TS=(25(OH)D3) OR TS=(“25(OH) D3”) OR TS=(“25 (OH) D3”) OR TS=(“1, 25 dihydroxy vitamin D 3”) OR TS=(calcifediol) OR TS=(calcitriol) OR TS=(Calciol) OR TS=(“Vitamin D 3”) OR TS=(“Vitamin D3”) OR TS=(Cholecalciferol*) OR TS=(“Hydroxyvitamins D”) OR TS=(Hydroxycholecalciferol) OR TS=(Calciferol*) OR TS=(“Vitamin D 2”) OR TS=(“Vitamin D2”) OR TS=(Ergocalciferol) OR TS=(“25 Hydroxyvitamin D 2”) OR TS=(“25 Hydroxyergocalciferol”) OR TS=(“25-Hydroxyvitamin D2”) OR TS=(“25 Hydroxyvitamin D2”) OR TS=(25-Hydroxycalciferol) OR TS=(“25 Hydroxycalciferol”) OR TS=(“(3 beta,5Z,7E)-9,10-Secocholesta-5,7,10(19)-trien-3-ol”) OR TS=(Calciferols) OR TS=(“25-hydroxyvitamin D”) OR TS=(“24,25 dihydroxyvitamin D”) OR TS=(“25 hydroxyvitamin D”) OR TS=(“9,10 secocholesta 5,7,10(19) trien 23 yne 1,3,25 triol”) OR TS=(“9,10 secocholesta 5,7,10(19) trien 23 yne 3,25 diol”) OR TS=(“9,10 secocholesta 5,7,10(19),16 tetraen 23 yne 1,3,25 triol”) OR TS=(“9,10 secocholesta 5,7,10(19),22 tetraene 1,3,25,26 tetrol”) OR TS=(“ascorbic acid plus fluoride plus retinol plus vitamin D”) OR TS=(“calcium carbonate plus ferrous fumarate plus vitamin D”) OR TS=(“calcium phosphate dibasic plus ferrous sulfate plus manganese sulfate plus nicotinic acid plus riboflavin plus thiamine plus vitamin D”) OR TS=(“9,10-Secoergosta-5,7,10(19),22-tetraene-3 beta,25-diol”) OR TS=(Ercalcidiol) OR TS=(Tachystin) OR TS=(Dihydrotachysterin) OR TS=(Calcamine))

**The search terms for Scopous was as follow:**

( TITLE-ABS-KEY ( pregnan* )  OR  TITLE-ABS-KEY ( gestation )  OR  TITLE-ABS-KEY ( "child bearing" )  OR  TITLE-ABS-KEY ( childbearing )  OR  TITLE-ABS-KEY ( gravidity )  OR  TITLE-ABS-KEY ( "intrauterine pregnancy" )  OR  TITLE-ABS-KEY ( "labor presentation" )  OR  TITLE-ABS-KEY ( "labour presentation" )  OR  TITLE-ABS-KEY ( "pregnancy maintenance" )  OR  TITLE-ABS-KEY ( "pregnancy trimesters" )  AND  TITLE-ABS-KEY ( "vitamin d" )  OR  TITLE-ABS-KEY ( 25  ( oh )  d )  OR  TITLE-ABS-KEY ( "25(OH) D" )  OR  TITLE-ABS-KEY ( "25 (OH) D" )  OR  TITLE-ABS-KEY ( "25 hydroxy vitamin D 3" )  OR  TITLE-ABS-KEY ( 25  ( oh )  d3 )  OR  TITLE-ABS-KEY ( "25(OH) D3" )  OR  TITLE-ABS-KEY ( "25 (OH) D3" )  OR  TITLE-ABS-KEY ( "1, 25 dihydroxy vitamin D 3" )  OR  TITLE-ABS-KEY ( calcifediol )  OR  TITLE-ABS-KEY ( calcitriol )  OR  TITLE-ABS-KEY ( calciol )  OR  TITLE-ABS-KEY ( "Vitamin D 3" )  OR  TITLE-ABS-KEY ( "Vitamin D3" )  OR  TITLE-ABS-KEY ( cholecalciferol* )  OR  TITLE-ABS-KEY ( "Hydroxyvitamins D" )  OR  TITLE-ABS-KEY ( hydroxycholecalciferol )  OR  TITLE-ABS-KEY ( calciferol* )  OR  TITLE-ABS-KEY ( "Vitamin D 2" )  OR  TITLE-ABS-KEY ( "Vitamin D2" )  OR  TITLE-ABS-KEY ( ergocalciferol )  OR  TITLE-ABS-KEY ( "25 Hydroxyvitamin D 2" )  OR  TITLE-ABS-KEY ( "25 Hydroxyergocalciferol" )  OR  TITLE-ABS-KEY ( "25-Hydroxyvitamin D2" )  OR  TITLE-ABS-KEY ( "25 Hydroxyvitamin D2" )  OR  TITLE-ABS-KEY ( 25-hydroxycalciferol )  OR  TITLE-ABS-KEY ( "25 Hydroxycalciferol" )  OR  TITLE-ABS-KEY ( "(3 beta,5Z,7E)-9,10-Secocholesta-5,7,10(19)-trien-3-ol" )  OR  TITLE-ABS-KEY ( calciferols )  OR  TITLE-ABS-KEY ( "25-hydroxyvitamin D" )  OR  TITLE-ABS-KEY ( "25 hydroxyvitamin D" )  OR  TITLE-ABS-KEY ( "9,10 secocholesta 5,7,10(19) trien 23 yne 1,3,25 triol" )  OR  TITLE-ABS-KEY ( "9,10 secocholesta 5,7,10(19) trien 23 yne 3,25 diol" )  OR  TITLE-ABS-KEY ( "9,10 secocholesta 5,7,10(19),16 tetraen 23 yne 1,3,25 triol" )  OR  TITLE-ABS-KEY ( "9,10 secocholesta 5,7,10(19),22 tetraene 1,3,25,26 tetrol" )  OR  TITLE-ABS-KEY ( "ascorbic acid plus fluoride plus retinol plus vitamin D" )  OR  TITLE-ABS-KEY ( "calcium carbonate plus ferrous fumarate plus vitamin D" )  OR  TITLE-ABS-KEY ( "calcium phosphate dibasic plus ferrous sulfate plus manganese sulfate plus nicotinic acid plus riboflavin plus thiamine plus vitamin D" )  OR  TITLE-ABS-KEY ( "9,10-Secoergosta-5,7,10(19),22-tetraene-3 beta,25-diol" )  OR  TITLE-ABS-KEY ( ercalcidiol )  OR  TITLE-ABS-KEY ( tachystin )  OR  TITLE-ABS-KEY ( dihydrotachysterin )  OR  TITLE-ABS-KEY ( calcamine ) )

**The search terms for Cochrane was as follow:**

("pregnancy"):ti,ab,kw AND ("vitamin D"):ti,ab,kw OR ("25-hydroxy vitamin D"):ti,ab,kw OR ("25-OH D"):ti,ab,kw OR ("25-OH-D"):ti,ab,kw

Supplementary Table 1. Sub-group analysis of the effect of vitamin D supplementation on the level of 25(OH)D

| **Biomarkers** | **No of trials** | **SMD (95% CI)** | **I^2^** | **P Heterogeneity** | **between-study variance Tau-squared** |
| --- | --- | --- | --- | --- | --- |
| **25(OH)D** | 15 | 2.07 (1.51, 2.63) | 94.1% | <0.001 | 1.08 |
| **Baseline vitamin D status** |  | | | | |
| **Deficient** | 10 | 2.48 (1.69, 3.27) | 94.7% | <0.001 | 1.46 |
| **Insufficient** | 4 | 1.35 (0.49, 2.21) | 91.6% | <0.001 | 0.68 |
| **Sufficient** | 1 | 1.39 (0.57, 2.21) | - | - | 0.00 |
| **Duration**  **(week)** |  | | | | |
| **<15** | 8 | 2.93 (1.81, 4.04) | 95.3% | <0.001 | 2.39 |
| **>15** | 7 | 1.24 (0.79, 1.70) | 86.2% | <0.001 | 0.30 |
| **Dose of vitamin D supplementation in intervention group (IU/d)** |  | | | | |
| **<1000** | 2 | 2.51 (-0.74, 5.76) | 96.8% | <0.001 | 5.34 |
| **1000-2000** | 5 | 1.12 (0.92, 1.37) | 0.0% | 0.58 | 0.00 |
| **2000-4000** | 6 | 3.11 (1.74, 4.48) | 95.6% | <0.001 | 2.72 |
| **>4000** | 2 | 1.71 (-0.68, 4.11) | 97.0% | <0.001 | 2.91 |
| **vitamin D supplementation in control group** |  | | | | |
| **Yes** | 10 | 1.24 (0.62, 1.87) | 89.5% | <0.001 | 0.43 |
| **No** | 4 | 1.37 (0.54, 2.21) | 94.4% | <0.001 | 1.63 |
| **Health status** |  | | | | |
| **Healthy** | 9 | 1.49 (0.94, 2.04) | 91.3% | <0.001 | 0.53 |
| **Pregnancy complications** | 6 | 3.09 (1.75, 4.44) | 95.7% | <0.001 | 2.62 |
| **Gestational age at first visit (week)** |  | | | | |
| **<12** | 2 | 0.63 (0.32, 0.93) | 41.0% | 0.19 | 0.02 |
| **12-24** | 4 | 1.60 (0.69, 2.4) | 80.1% | <0.001 | 0.49 |
| **>24** | 9 | 2.73 (1.76, 3.62) | 94.7% | <0.001 | 2.01 |
| **Co-supplementation** |  | | | | |
| **Yes** | 6 | 2.34 (1.24, 3.44) | 93.8% | <0.001 | 1.72 |
| **No** | 9 | 1.93 (1.26, 2.60) | 94.3% | <0.001 | 0.93 |
| **Overall risk of bias** |  | | | | |
| **Low risk of bias** | 6 | 1.79 (1.12, 2.47) | 89.0% | <0.001 | 0.60 |
| **Some concerns** | 6 | 2.72 (1.36, 4.08) | 96.8% | <0.001 | 2.70 |
| **High risk of bias** | 3 | 1.65 (0.62, 2.68) | 90.2% | <0.001 | 0.73 |

Supplementary Table 2. Sub-group analysis of the effect of vitamin D supplementation on hs-CRP level

| **Biomarkers** | **No. of trials** | **SMD (95% CI)** | **I^2^** | **P Heterogeneity** | **between-study variance Tau-squared** |
| --- | --- | --- | --- | --- | --- |
| **hs-CRP** | 11 | 0.24 (-0.55, 1.03) | 95.4% | <0.001 | 1.65 |
| **Baseline vitamin D status** |  | | | | |
| **Deficient** | 10 | 0.33 (-0.55, 1.22) | 95.8% | <0.001 | 1.86 |
| **Insufficient** | 1 | -0.31 (-0.85, 0.22) | - | - | 0.00 |
| **Sufficient** | 0 | - | - | - | - |
| **Duration (week)** |  | | | | |
| **<15** | 9 | 0.12 (-0.78, 1.04) | 95.4% | <0.001 | 1.77 |
| **>15** | 2 | 0.80 (-0.66, 2.27) | 94.0% | <0.001 | 1.05 |
| **Dose of vitamin D supplementation in intervention group (IU/d)** |  | | | | |
| **<1000** | 2 | -2.03 (-5.40, 1.32) | 95.4% | <0.001 | 5.74 |
| **1000-2000** | 5 | -0.07 (-0.43, 0.27) | 11.7% | 0.28 | 0.007 |
| **2000-4000** | 6 | -0.10 (-0.74, 0.54) | 89.1% | <0.001 | 0.57 |
| **>4000** | 1 | 14.36 (11.69, 17.02) | - | - | 0.00 |
| **vitamin D supplementation in control group** |  | | | | |
| **Yes** | 10 | 0.07 (-0.35, 0.50) | - | - | 0.00 |
| **No** | 1 | 0.31 (-0.59, 1.22) | 95.8% | <0.001 | 1.96 |
| **Health status** |  | | | | |
| **Healthy** | 3 | -0.67 (-3.02, 1.67) | 97.7% | <0.001 | 4.18 |
| **Pregnancy complications** | 8 | 0.47 (-0.37, 1.32) | 94.4% | <0.001 | 1.32 |
| **Gestational age at first visit (week)** |  | | | | |
| **<12** | 1 | 0.07 (-0.35, 0.50) | - | - | - |
| **12-24** | 0 | - | - | - | - |
| **>24** | 10 | 0.31 (-0.59, 1.22) | 95.8% | <0.001 | 1.96 |
| **Co-supplementation** |  | | | | |
| **Yes** | 4 | 0.05 (-0.93, 1.03) | 93.0% | <0.001 | 0.94 |
| **No** | 7 | 0.54 (-0.67, 1.76) | 96.5% | <0.001 | 2.46 |
| **Overall risk of bias** |  | | | | |
| **Low risk of bias** | 4 | -1.04 (-2.20, 0.12) | 93.9% | <0.001 | 1.03 |
| **Some concerns** | 3 | 0.04 (-1.41, 1.50) | 95.4% | <0.001 | 1.59 |
| **High risk of bias** | 4 | 2.43 (0.52, 4.33) | 97.4% | <0.001 | 3.38 |

Supplementary Table 3. Sub-group analysis of the effect of vitamin D supplementation on the TAC level

| **Biomarkers** | **No of trials** | **SMD (95% CI)** | **I^2^** | **P Heterogeneity** | **between-study variance Tau-squared** |
| --- | --- | --- | --- | --- | --- |
| **TAC** | 9 | 2.13 (1.04, 3.22) | 96.6% | <0.001 | 2.65 |
| **Baseline vitamin D status** |  | | | | |
| **Deficient** | 9 | 2.13 (1.04, 3.22) | 96.6% | <0.001 | 2.65 |
| **Insufficient** | 0 | - | - | - | - |
| **Sufficient** | 0 | - | - | - | - |
| **Duration (week)** |  | | | | |
| **<15** | 7 | 2.71 (1.19, 4.23) | 97.2% | <0.001 | 0.00 |
| **>15** | 2 | 0.32 (-0.01, 0.64) | 0.0% | 0.39 | 4.033 |
| **Dose of vitamin D supplementation in intervention group (IU/d)** |  | | | | |
| **<1000** | 2 | 3.34 (-2.01, 8.69) | 98.5% | <0.001 | 14.68 |
| **1000-2000** | 2 | 3.04 (-2.58, 8.68) | 98.7% | <0.001 | 16.31 |
| **2000-4000** | 4 | 0.49 (0.01, 0.98) | 98.5% | <0.001 | 14.68 |
| **>4000** | 1 | 5.40 (4.29, 6.51) | - | - | 0.00 |
| **vitamin D supplementation in control group** |  | | | | |
| **Yes** | 8 | 0.20(-0.22, 0.63) | - | - | 0.00 |
| **No** | 1 | 2.40 (1.12, 3.68) | 96.8% | <0.001 | 3.24 |
| **Health status** |  | | | | |
| **Healthy** | 3 | 0.41 (0.13, 0.68) | 0.0% | 0.41 | 0.00 |
| **Pregnancy complications** | 6 | 3.09 (1.20, 4.98) | 97.6% | <0.001 | 5.36 |
| **Gestational age at first visit (week)** |  | | | | |
| **<12** | 1 | 0.20 (-0.22, 0.63) | - | - | 0.00 |
| **12-24** | 0 | - | - | - | - |
| **>24** | 8 | 2.40 (1.12, 3.68) | 96.8% | <0.001 | 3.24 |
| **Co-supplementation** |  | | | | |
| **Yes** | 4 | 0.53 (0.03, 1.02) | 71.5% | 0.015 | 0.18 |
| **No** | 5 | 3.57 (1.21, 5.94) | 98.0% | <0.001 | 7.04 |
| **Overall risk of bias** |  | | | | |
| **Low risk of bias** | 1 | - | - | - | 0.00 |
| **Some concerns** | 4 | -0.11(-0.63, 0.41) | 15.8% | 0.31 | 0.01 |
| **High risk of bias** | 4 | 4.39 (0.73, 8.04) | 98.4% | <0.001 | 13.63 |

Supplementary Table 4. Sub-group analysis of the effect of vitamin D supplementation on the MDA level

| **Biomarkers** | **No. of trials** | **SMD (95% CI)** | **I^2^** | **P Heterogeneity** | **between-study variance Tau-squared** |
| --- | --- | --- | --- | --- | --- |
| **MDA** | 6 | -0.46 (-0.87, -0.05) | 74.7% | 0.001 | 0.19 |
| **Baseline vitamin D status** |  | | | | |
| **Deficient** | 6 | -0.46 (-0.87, -0.05) | 74.7% | 0.001 | 0.19 |
| **Insufficient** | 0 | - | - | - | - |
| **Sufficient** | 0 | - | - | - | - |
| **Duration (week)** |  | | | | |
| **<15** | 4 | -0.73 (-1.00, -0.47) | 0.0% | 0.80 | 0.00 |
| **>15** | 2 | 0.058 (-0.58, 0.70) | 73.5% | 0.05 | 0.16 |
| **Dose of vitamin D supplementation in intervention group (IU/d)** |  | | | | |
| **<1000** | 1 | -0.74 (-1.26, -0.22) | - | - | 0.00 |
| **1000-2000** | 1 | 0.37 (-0.05, 0.80) | - |  | 0.00 |
| **2000-4000** | 4 | -0.61 (-0.88, -0.34) | 5.4% | 0.36 | 0.004 |
| **>4000** | 0 | - | - | - | - |
| **vitamin D supplementation in control group** |  | | | | |
| **Yes** | 1 | 0.37 (-0.05, 0.80) | - | - | 0.00 |
| **No** | 5 | -0.64 (-0.87, -0.40) | 0.0% | 0.50 | 0.00 |
| **Health status** |  | | | | |
| **Healthy** | 3 | -0.205 (-0.86, 0.45) | 81.6% | 0.004 | 0.27 |
| **Pregnancy complications** | 3 | -0.73 (-1.03, -0.42) | 0.0% | 0.60 | 0.00 |
| **Gestational age at first visit (week)** |  | | | | |
| **<12** | 1 | 0.37 (-0.05, 0.80) | - | - | 0.00 |
| **12-24** | 0 | - |  |  |  |
| **>24** | 5 | -0.64 (-0.87, -0.40) | 0.0% | 0.5 | 0.00 |
| **Co-supplementation** |  | | | | |
| **Yes** | 4 | -0.63 (-0.91, -0.35) | 10.4% | 0.34 | 0.008 |
| **No** | 2 | -0.13 (-1.15, 0.88) | 89.0% | 0.003 | 0.48 |
| **Overall risk of bias** |  | | | | |
| **Low risk of bias** | 1 | -0.58 (-1.11, -0.04) | - | - | 0.00 |
| **Some concerns** | 4 | -0.65 (-0.92, 0.38) | 9.3% | 0.34 | 0.007 |
| **High risk of bias** | 1 | 0.37 (-0.05, 0.80) | - | - | 0.0 |

Supplementary Table 5. Sub-group analysis of the effect of vitamin D supplementation on the GSH level

| **Biomarkers** | **No. of trials** | **SMD (95% CI)** | **I^2^** | **P Heterogeneity** | **between-study variance Tau-squared** |
| --- | --- | --- | --- | --- | --- |
| **GSH** | 9 | 4.36 (2.90, 5.83) | 97.6% | <0.001 | 4.49 |
| **Baseline vitamin D status** |  | | | | |
| **Deficient** | 9 | 4.36 (2.90, 5.83) | 97.6% | <0.001 | 4.49 |
| **Insufficient** | 0 | - | - | - | - |
| **Sufficient** | 0 | - | - | - | - |
| **Duration (week)** |  | | | | |
| **<15** | 8 | 5.02 ( 3.29, 6.75) | 97.9% | <0.001 | 5.56 |
| **>15** | 1 | 0.93 (0.39, 1.46) | - | - | 0.00 |
| **Dose of vitamin D supplementation in intervention group (IU/d)** |  | | | | |
| **<1000** | 2 | 5.35 (-4.92, 15.62) | 99.0% | <0.001 | 54.41 |
| **1000-2000** | 2 | 8.59 (-4.23, 21.43) | 98.8% | <0.001 | 84.73 |
| **2000-4000** | 4 | 0.63 (0.37, 0.90) | 1.9% | 0.38 | 0.001 |
| **>4000** | 1 | 18.41 (15.02, 21.80) | - | - | 0.00 |
| **vitamin D supplementation in control group** |  |  |  |  |  |
| **Yes** | 1 | 2.12 (1.56, 2.68) | - | - | 0.00 |
| **No** | 8 | 4.80 (3.13, 6.47) | 97.7% | <0.001 | 5.17 |
| **Health status** |  | | | | |
| **Healthy** | 2 | 0.53 (-0.21, 1.29) | 76.4% | 0.039 | 0.228 |
| **Pregnancy complications** | 7 | 5.99 (3.91, 8.07) | 98.1% | <0.001 | 7.12 |
| **Gestational age at first visit (week)** |  | | | | |
| **<12** | 0 | - | - | - | - |
| **12-24** | 0 | - | - | - | - |
| **>24** | 9 | 4.36 (2.9, 5.83) | 97.6% | <0.001 | 4.49 |
| **Co-supplementation** |  | | | | |
| **Yes** | 5 | 8.93 (5.10, 12.77) | 37.9% | 0.18 | 0.04 |
| **No** | 4 | 11.06 (1.88, 20.24) | 99.0% | <0.001 | 86.26 |
| **Overall risk of bias** |  | | | | |
| **Low risk of bias** | 1 | 0.57 (0.03, 1.10) | - | - | 0.00 |
| **Some concerns** | 4 | 0.53 (0.17, 0.89) | 48.1% | 0.12 | 0.06 |
| **High risk of bias** | 4 | 11.50 (3.53, 19.47) | 98.6% | <0.001 | 64.65 |

*Supplementary* Table 6. The results of meta-regression of RCTs evaluating the effect of vitamin D supplementation on the levels of 25(OH)D, hs-CRP, MDA, TAC and GSH

| **Marker** | **Variables** | **Coefficient** | **SE** | **t** | **P** | **95% CI** | | **tau^2^** | **I^2^ residual** | **Adjusted R^2^** |
| --- | --- | --- | --- | --- | --- | --- | --- | --- | --- | --- |
|  |  |  |  |  |  | **LL** | **UL** |  |  |  |
| **25(OH)D** | Duration of supplementation | -0.08 | 0.05 | 1.45 | 0.17 | -0.20 | 0.04 | 3.27 | 93% | 7.68% |
|  | Dose of supplementation | 0.0003 | 0.0003 | 0.87 | 0.4 | -0.0004 | 0.001 | 3.63 | 94.49% | -2.61% |
|  | Gestational age at the beginning of the study | 0.10 | 0.09 | 1.02 | 0.33 | -0.11 | 0.32 | 3.91 | 93.67% | -0.33% |
|  | Sample size | -0.01 | 0.01 | -1.14 | 0.27 | -0.05 | 0.01 | 3.91 | 93.67% | -0.33% |
| **CB 25(OH)D** | Duration of supplementation | -0.19 | 0.02 | 6.99 | 0.09 | -0.53 | 0.15 | 0 | 0.00% | 100% |
|  | Dose of supplementation | 0.0006 | 0.0004 | 1.50 | 0.37 | -0.004 | 0.006 | 0.81 | 91.52% | 40.17% |
|  | Gestational age at the beginning of the study | 0.13 | 0.02 | 6.99 | 0.09 | -0.10 | 0.37 | 0 | 0.00% | 100% |
|  | Sample size | 0.02 | 0.12 | 0.15 | 0.90 | -1.59 | 1.63 | 2.70 | 97.95% | -98.68% |
| **hs-CRP** | Duration of supplementation | -0.02 | 0.22 | 0.07 | 0.95 | -0.53 | 0.50 | 21.42 | 95.74% | -13.05% |
|  | Dose of supplementation | 0.001 | 0.0009 | 1.34 | 0.21 | -0.0009 | 0.004 | 17.45 | 95.67% | 7.89% |
|  | Gestational age at the beginning of the study | 0.07 | 0.32 | 0.23 | 0.83 | -0.65 | 0.80 | 21.31 | 95.81% | -12.43% |
|  | Sample size | -0.15 | 0.23 | 0.66 | 0.52 | -0.69 | 0.37 | 20.41 | 95.82% | -7.71% |
| **TAC** | Duration of supplementation | -0.08 | 0.13 | 0.61 | 0.56 | -0.39 | 0.23 | 7.56 | 96.86% | -9.24% |
|  | Dose of supplementation | -0.0004 | 0.0006 | 0.75 | 0.48 | -0.002 | 0.001 | 7.36 | 96.94% | -6.33% |
|  | Gestational age at the beginning of the study | 2.65 | 1.74 | 1.52 | 0.17 | -1.47 | 6.78 | 5.95 | 96.66% | 14.01% |
|  | Sample size | 0.14 | 0.18 | 0.78 | 0.46 | -0.29 | 0.58 | 7.32 | 96.82% | -5.76% |
| **MDA** | Duration of supplementation | 0.05 | 0.01 | 4.30 | 0.01 | 0.08 | 0.08 | 0 | 0.00% | 100% |
|  | Dose of supplementation | -0.00004 | 0.0001 | 0.29 | 0.79 | -0.0005 | 0.0004 | 0.223 | 79.03% | -27.42% |
|  | Gestational age at the beginning of the study | -0.53 | 0.35 | 1.51 | 0.20 | -1.53 | 0.45 | 0.12 | 66.37% | 29.01% |
|  | Sample size | -0.06 | 0.01 | 4.05 | 0.02 | -0.10 | -0.02 | 0 | 0.00% | 100% |
| **GSH** | Duration of supplementation | 0.87 | 1.31 | 0.66 | 0.52 | -2.23 | 3.97 | 54.13 | 97.84% | -8.23% |
|  | Dose of supplementation | -0.0001 | 0.001 | -0.06 | 0.95 | -0.004 | 0.003 | 57.51 | 97.83% | -14.99% |
|  | Gestational age at the beginning of the study | -3.62 | 8.00 | -0.45 | 0.66 | -22.56 | 15.30 | 55.99 | 97.75% | -11.96% |
|  | Sample size | -0.99 | 0.36 | -2.71 | 0.03 | -1.87 | -0.12 | 27.39 | 97.86% | 45.22% |
| Abbreviations: 25(OH)D: 25-​hydroxyvitamin D3, CB: Cord Blood, CI: confidence interval, hs-CRP: high-sensitive C-reactive protein, TAC: Total antioxidant capacity, MDA: Malondialdehyde, GSH: Glutathione, SE: Standard error | | | | | | | | | | |
